# A Theoretical Framework for a Mathematical Cognitive Model for Adaptive Learning Systems

**DOI:** 10.3390/bs13050406

**Published:** 2023-05-12

**Authors:** Siyu Sun, Xiaopeng Wu, Tianshu Xu

**Affiliations:** 1 College of Elementary Education, Capital Normal University, Beijing 100048, China; sysun@cnu.edu.cn; 2 Faculty of Education, Northeast Normal University, Changchun 130024, China; 3 College of Teacher Education, East China Normal University, Shanghai 200062, China; xvtianshu@163.com

**Keywords:** cognitive model, mathematical learning, adaptive learning system, interpretive structural modeling

## Abstract

The emergence of artificial intelligence has made adaptive learning possible, but building an adaptive system requires a comprehensive understanding of students’ cognition. The cognitive model provides a crucial theoretical framework to explore students’ cognitive attributes, making it vital for learning assessment and adaptive learning. This study investigates 52 experts, including primary and secondary school teachers, mathematics education experts, and graduate students, based on the 16 cognitive attributes in the TIMSS 2015 assessment framework. Through an analysis of their attribute questionnaires, the Interpretive structural modeling (ISM) method is used to construct a five-level mathematical cognitive model. The model is then revised through oral reports and expert interviews, resulting in a final cognitive model ranging from “memorize” to “justify”. The cognitive model describes the relationship between different attributes in detail, enabling the development of adaptive systems and aiding in the diagnosis of students’ cognitive development and learning paths in mathematics.

## 1. Introduction

The importance of mathematics in human development has been widely recognized, as many key competencies are closely related to learning mathematics, such as creative thinking [1], critical thinking [2,3], and design thinking [4]. However, how mathematical thinking may be effectively assessed is still an urgent issue to be addressed. With the evolution of education, the goal of assessment has evolved from checking the effects of student learning to the promotion of students learning more effectively [5,6,7] (pp. 19–39). Traditional measurements normally focus on the evaluation results rather than the process because the thought process during an exam cannot be directly observed and evaluated. Only through classroom observation and interviews can we obtain a formative evaluation of students [8]. However, there are some problems in evaluating students via classroom observation and interview, such as complex evaluation processes, high requirements for the quality of evaluators, poor consistency of evaluation results, and a lack of ability to carry out a large-scale evaluation. Therefore, the means of personalizing learning and evaluation have attracted an increasing amount of attention from education measurement researchers [9,10].

With the advancement of measurement theory, a new generation of assessment theory—cognitive diagnostic assessment—has emerged. In a cognitive diagnostic assessment, which is based on the characteristics of a psychological evaluation, detailed information about a student’s diagnosis can be obtained through items with a cognitive diagnosis function and the analysis of a mathematical model, which provides the possibility for a personalized evaluation and adaptive learning [11,12]. As part of a new generation of evaluation theories, cognitive diagnosis assessment aims to infer learners’ mastery of cognitive skills by using cognitive science and statistical models, further constructing fine-grained reports and providing formative feedback [13,14,15].

As an important theoretical basis for guiding the development of cognitive diagnosis assessment, the cognitive model plays a leading role in designing the structure of a cognitive diagnosis assessment, the construction of attribute relationships, and the development of evaluation questionnaires. High-quality cognitive models necessitate collaboration between pedagogical professionals, psychologists, measurement scientists, and teachers. However, there is no well-established methodology for developing cognitive models. The cognitive models that are usually based on the expert method, student verbal reports, and the methods in the literature and have low consistency and lack operability [16]. Therefore, it is critical to investigate new cognitive model construction approaches and construct well-structured and operational cognitive models.

## 2. Literature Review

### 2.1. The Cognitive Model and Its Construction

The term “cognitive model” originated in the field of computer science, and it refers to the process of modeling human problem solving and mental task processing. This concept is commonly thought to be a computational model used to simplify the explanation of human problem solving in cognitive psychology, which is supported by research on human cognitive processing [17] (pp. 433–439). The cognitive model (CM) is commonly defined in educational research as “used to describe the cognitive process of students’ problem-solving in the test”. It aids in the identification of students’ various learning levels in terms of their knowledge and skills, as well as providing an explanation for and prediction of student performance [18] (p. 56). The cognitive model not only includes comprehensive cognitive attributes but also establishes the underlying operational relationship between them. The cognitive model serves as a precondition as well as an optimal measurement model for cognitive diagnostic assessment [19]. It also provides a guarantee for the realization of a structured evaluation.

There are two main ways of constructing a cognitive model. The first is known as the “verbal report”, which is based on students’ cognitive models. It mainly conducts interviews with students of different levels to understand their problem-solving process. For example, Gierl recorded the response processes of students who participated in the “think-aloud” seminar and utilized a flowchart to transcribe their answering process, thereby constructing a student cognitive model [20]. The other is the “expert-based cognitive model”, which mainly forms cognitive attributes based on theories and then determines the hierarchical relationships between attributes through interviews with subject experts. The expert model is more in line with average students and can more accurately anticipate most students’ cognitive processes [21]. In general, regardless of the approach used to construct the cognitive model, the attributes in the model and the relationships between them should effectively reflect the probable cognitive process of respondents when answering questions. Only an accurate cognitive model can provide a solid foundation for estimating students’ learning states.

### 2.2. Cognitive Attributes and TIMSS Cognitive Framework

Determining cognitive attributes is the basis of constructing a cognitive model and is also a fundamental link in cognitive diagnostic assessment. Some researchers believe that cognitive attributes are used to describe the procedures, skills, strategies, and knowledge that students must have in the process of problem-solving [22,23], while others assert that cognitive attributes refer to the basic cognitive processes and skills in problem solving [24]. Reasonable cognitive attributes must also consider the fine granularity of attribute differentiation. When the granularity is small, the process described by the cognitive model will be more refined, but the model will also become more complicated. Since the effectiveness of the cognitive model and the reliability of a cognitive diagnosis both depend on the quality of the cognitive attributes, the importance of determining reasonable cognitive attributes is self-evident [25].

The Trends in International Mathematics and Science Study (TIMSS) is the largest international education assessment, involving more than 60 countries or regions around the world [26] (pp. 116–124) [27] (pp. 19–20). The TIMSS has a broad spectrum of impact. Some policymakers refer to the test results to formulate new education policies, while some education researchers improve curricula and teaching based on the survey results [28]. The test evaluates students’ mathematical abilities in terms of both content and cognition. The cognitive framework consists of three domains of “understanding, application, and reasoning”, and can be further refined into 16 attributes [29]. There are many similarities between the three-domain theoretical framework of TMISS and Bloom’s taxonomy. Bloom’s taxonomy is a multi-tiered model of classifying thinking according to six cognitive levels of complexity. It was proposed by Bloom around 1950 and has been continuously revised. The revised cognitive objective theory includes six levels: “remembering, understanding, applying, analyzing, evaluating, and creating” [30]. This theory has been applied in teaching and assessment across many disciplines, such as mathematics education [31] and science education [32]. As the TIMSS does not examine pupils’ cognitive abilities, only the first-level indications are compared internationally. The cognitive model, on the other hand, has stricter criteria for fine-grained qualities, and a sufficient number of attributes can more accurately represent students’ cognitive processes. The cognitive framework of the TIMSS is based on the mathematics curriculum standards of various countries and the students’ mathematical abilities. Furthermore, numerous cognitive diagnostic studies are based on the TIMSS [33], demonstrating its high credibility and effectiveness.

### 2.3. Interpretive Structural Modeling (ISM)

When a complex system contains complex elements, the system cannot be seen and defined clearly, so it is very necessary to structure the system through the use of a method. Interpretive structural modeling (ISM) is an ideal method for dealing with complex relational systems. A comprehensive system model is produced using this technology which precisely depicts complicated situations by combining a number of different directly and indirectly relevant components [34,35,36]. An ambiguous system can be transformed into a visible and well-defined system simply through interpretive structural modeling. Recently, interpretive structural modeling has been employed in a variety of sectors, including educational research [37]. For example, Debnath et. al proposed an improved method for technical education through an interpretive structural model. This research can assist educators in developing curricula and improving teaching methods [38].

The cognitive framework of the TIMSS only contains attributes. In addition to cognitive attributes, a complete cognitive model should also reflect the hierarchical and sequential links between them, to more accurately describe the thinking process of students when problem solving. The differentiation of cognitive attributes in the TIMSS has high granularity, but it does not investigate the links between the attributes or how to develop a cognitive model. Therefore, the interpretive structural model can be used to synthesize the opinions of experts, construct the complex links between the cognitive attributes, and form a cognitive model with a high level of efficiency and strong operability.

The application of cognitive diagnostic assessment is vital for large-scale individualized evaluations and adaptive learning, and the efficacy of a cognitive diagnostic evaluation is dependent on a logical and highly operable cognitive model [39]. The expert method, verbal report, and the literature method are the most common cognitive model construction approaches. These methods guarantee the effectiveness of the cognitive model from the perspective of validity. However, because methods such as expert interviews and student reports are highly subjective, there are significant disparities among experts. The construction of a cognitive model is a very complex procedure, so there are few cognitive models that can be directly applied to cognitive diagnosis in education. In recent years, interpretive structural modeling has been applied to education research [40,41]. It primarily quantifies qualitative research and investigates the logical relationships between diverse aspects, resulting in a novel concept for cognitive model development. Through the interpretive structural model, the hierarchical relationship and causality among cognitive attributes can be determined, and the roles of each attribute in the system can be analyzed. Therefore, this study attempts to construct a mathematical cognitive model through the interpretive structural model, with the goal of providing a theoretical foundation for the development of adaptive systems, mathematics curricula, teaching, and cognitive diagnostic assessments.

## 3. Methods

### 3.1. Sample

The cognitive model construction in this study involved two stages. In the first stage, 33 primary school mathematics teachers, 15 graduate students, and 4 mathematics education specialists in Shanghai were invited to evaluate the relationships between cognitive attributes from both practical and theoretical perspectives. They needed to judge the pair relationship between the 16 cognitive attributes in Table 1. In the second stage, six mathematics education professors were invited to revise the preliminary model, and a fourth-grade child was randomly selected to give an oral report to verify the efficacy of the cognitive model.

### 3.2. Interpretive Structural Modeling (ISM)

The interpretative structural model is an ideal method for dealing with a complex relational system and can combine related and unrelated elements in the system into a comprehensive model. The various steps involved in ISM are as follows:

Step 1: Set the key problem and determine the variables related to the problem.

Step 2: A structural self-interaction matrix (SSIM) indicating the pairwise relationship among these variables is developed.

Step 3: Transform the SSIM to a reachability matrix (RX).

Step 4: Hierarchically decompose the reachability matrix, link the relationship between variables, and form a structural model diagram.

Step 5: The ISM model is checked for conceptual inconsistency and necessary modifications.

**Figure 1:**
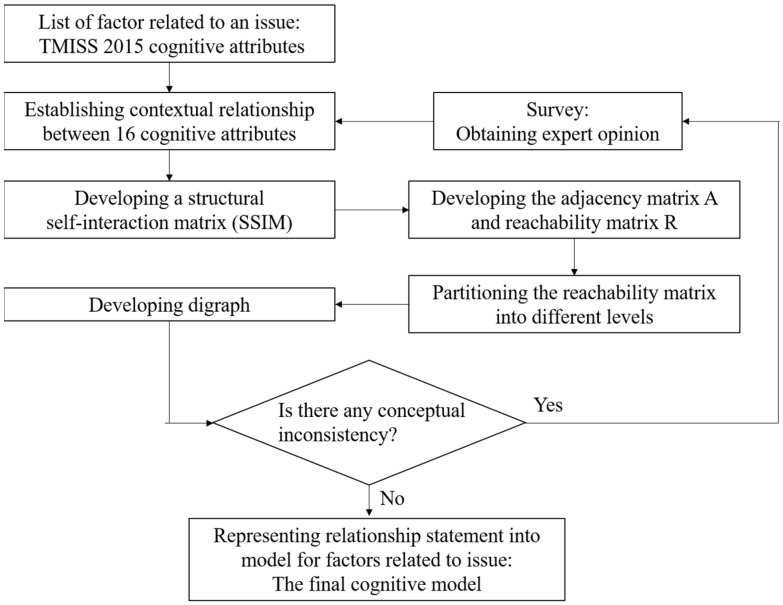
Flow diagram for constructing a cognitive model.

The focus of this study is on which cognitive attributes affect students’ success in solving mathematical problems. Various steps involved in constructing a cognitive model using ISM are illustrated in Figure 1.

In our study, 16 mathematical cognitive attributes corresponding to the problem variables are listed in Table 1. The structural self-intersection matrix and the reachability matrix were created after 52 experts completed the “cognitive attribute relationship” questionnaire. The reachability matrix was then hierarchically dissected, and the attributes’ correlations were linked to developing the cognitive model.

### 3.3. Data Collection

This study mainly used three types of data collection methods. The first was a questionnaire survey, which gave 52 experts 40 min to become familiar with the cognitive attributes before determining the pairwise associations of the attributes in the questionnaire. The group interview method then followed, which was primarily employed to modify the cognitive model. Following the creation of the conceptual framework based on the ISM, six mathematics education professionals were invited to have an open discussion on the constructed model and offer suggestions for its improvement. This discussion lasted a total of two hours. The last method was a verbal report, which asked students to make oral statements about their problem-solving processes, after which a comparative analysis was performed with the cognitive model to verify the effectiveness of the model. Two different types of questions from the TIMSS test were extracted for this study, and a fourth-grade student was asked to present her thoughts while reading the questions. The students’ problem-solving process was then depicted in a flowchart and confirmed using the developed cognitive model.

## 4. Procedure and Results

### 4.1. Constructing a Cognitive Model with the ISM

#### 4.1.1. Attributes and Their Relationships in Mathematical Cognitive Model

Since a cognitive attribute is the theoretical basis of a cognitive diagnosis assessment, the determination of a cognitive attribute should not only consider its own accuracy but also ensure that it can be related to the items. Among existing large-scale evaluations, the TIMSS is relatively well-established in terms of its cognitive assessment, and it evaluates students’ mathematical literacy from perspectives of both content and cognition, allowing the cognitive attributes to be properly matched with its items. Many researchers have conducted cognitive diagnosis research based on the TIMSS [42,43,44,45], which shows that the cognitive attributes of the TIMSS have good reliability and validity.

The first step in ISM is to identify the variables, which in this case, are the cognitive processes of students involved in solving mathematical problems. The cognitive attributes of the TIMSS include three aspects of “knowing, applying and reasoning”, and each aspect contains different attributes. Sixteen cognitive attributes were preliminarily generated by upgrading the cognitive process framework in the TIMSS 2015, as shown in Table 1.

The SSIM was developed based on the relationship between the variables. In the SSIM, V, A, X, and O are usually used to represent the interrelationship between factors. V means that i influences j, A means that j influences i, X means that two elements influence each other, and O means that i and j have no influence on each other. The determination of the relationships between cognitive attributes by experts was collected through 52 questionnaires, based on which the SSIM of the mathematical cognitive attributes was formed according to the standard of 50% (i.e., more than 25 experts believe that there is a relationship between the two attributes), as shown in Table 2.

#### 4.1.2. Adjacency Matrix and Reachability Matrix of Cognitive Attributes

The adjacency matrix is a matrix also used to describe the relationships between elements. The relationship between the two factors S_i_ and S_j_ is represented by the matrix a_ij_. When the factor S_i_ has a direct influence on S_j_, a_ij_ = 1; when the factor S_i_ has no direct influence on S_j_, a_ij_ = 0. Therefore, the adjacency matrix *A* in Equation (1) can be constructed according to the SSIM.

(1) $$A=\left(\begin{array}{cccccccccccccccc} 0 & 1 & 1 & 1 & 1 & 1 & 1 & 0 & 0 & 0 & 0 & 0 & 0 & 0 & 0 & 0\\ 1 & 0 & 1 & 1 & 0 & 1 & 1 & 0 & 0 & 0 & 0 & 0 & 0 & 0 & 0 & 0\\ 0 & 0 & 0 & 0 & 0 & 0 & 0 & 0 & 0 & 0 & 0 & 0 & 0 & 0 & 0 & 0\\ 0 & 0 & 0 & 0 & 0 & 0 & 0 & 0 & 0 & 0 & 0 & 0 & 0 & 0 & 0 & 0\\ 0 & 0 & 0 & 0 & 0 & 0 & 0 & 0 & 0 & 0 & 0 & 0 & 0 & 0 & 0 & 0\\ 0 & 0 & 0 & 0 & 0 & 0 & 0 & 1 & 1 & 1 & 1 & 1 & 0 & 1 & 0 & 0\\ 0 & 0 & 0 & 0 & 0 & 0 & 0 & 0 & 0 & 0 & 0 & 0 & 0 & 0 & 0 & 0\\ 0 & 0 & 0 & 0 & 0 & 0 & 0 & 0 & 1 & 1 & 0 & 0 & 1 & 0 & 0 & 0\\ 0 & 0 & 0 & 0 & 0 & 0 & 0 & 0 & 0 & 0 & 0 & 0 & 0 & 0 & 0 & 0\\ 0 & 0 & 0 & 0 & 0 & 0 & 0 & 0 & 0 & 0 & 0 & 0 & 0 & 0 & 0 & 0\\ 0 & 0 & 0 & 0 & 0 & 0 & 0 & 0 & 0 & 0 & 0 & 1 & 1 & 1 & 1 & 1\\ 0 & 0 & 0 & 0 & 0 & 0 & 0 & 0 & 0 & 0 & 0 & 0 & 1 & 0 & 0 & 1\\ 0 & 0 & 0 & 0 & 0 & 0 & 0 & 0 & 0 & 0 & 0 & 0 & 0 & 0 & 0 & 0\\ 0 & 0 & 0 & 0 & 0 & 0 & 0 & 0 & 0 & 0 & 0 & 0 & 0 & 0 & 1 & 0\\ 0 & 0 & 0 & 0 & 0 & 0 & 0 & 0 & 0 & 0 & 0 & 0 & 0 & 0 & 0 & 0\\ 0 & 0 & 0 & 0 & 0 & 0 & 0 & 0 & 0 & 0 & 0 & 0 & 0 & 0 & 0 & 0 \end{array}\right)$$

The reachability matrix *R* in Equation (2) can be calculated iteratively by MATLAB. The reachability matrix *R* can show the elements with connection channels in the system, so the reachability matrix of cognitive attributes can clearly display the influence relationships between cognitive attributes.

(2) $$R=\left(\begin{array}{cccccccccccccccc} 1 & 1 & 1 & 1 & 1 & 1 & 1 & 1 & 1 & 1 & 1 & 1 & 1 & 1 & 1 & 1\\ 1 & 1 & 1 & 1 & 1 & 1 & 1 & 1 & 1 & 1 & 1 & 1 & 1 & 1 & 1 & 1\\ 0 & 0 & 1 & 0 & 0 & 0 & 0 & 0 & 0 & 0 & 0 & 0 & 0 & 0 & 0 & 0\\ 0 & 0 & 0 & 1 & 0 & 0 & 0 & 0 & 0 & 0 & 0 & 0 & 0 & 0 & 0 & 0\\ 0 & 0 & 0 & 0 & 1 & 0 & 0 & 0 & 0 & 0 & 0 & 0 & 0 & 0 & 0 & 0\\ 0 & 0 & 0 & 0 & 0 & 1 & 0 & 1 & 1 & 1 & 1 & 1 & 1 & 1 & 1 & 1\\ 0 & 0 & 0 & 0 & 0 & 0 & 1 & 0 & 0 & 0 & 0 & 0 & 0 & 0 & 0 & 0\\ 0 & 0 & 0 & 0 & 0 & 0 & 0 & 1 & 1 & 1 & 0 & 0 & 1 & 0 & 0 & 0\\ 0 & 0 & 0 & 0 & 0 & 0 & 0 & 0 & 1 & 0 & 0 & 0 & 0 & 0 & 0 & 0\\ 0 & 0 & 0 & 0 & 0 & 0 & 0 & 0 & 0 & 1 & 0 & 0 & 0 & 0 & 0 & 0\\ 0 & 0 & 0 & 0 & 0 & 0 & 0 & 0 & 0 & 0 & 1 & 1 & 1 & 1 & 1 & 1\\ 0 & 0 & 0 & 0 & 0 & 0 & 0 & 0 & 0 & 0 & 0 & 1 & 1 & 0 & 0 & 1\\ 0 & 0 & 0 & 0 & 0 & 0 & 0 & 0 & 0 & 0 & 0 & 0 & 1 & 0 & 0 & 0\\ 0 & 0 & 0 & 0 & 0 & 0 & 0 & 0 & 0 & 0 & 0 & 0 & 0 & 1 & 1 & 0\\ 0 & 0 & 0 & 0 & 0 & 0 & 0 & 0 & 0 & 0 & 0 & 0 & 0 & 0 & 1 & 0\\ 0 & 0 & 0 & 0 & 0 & 0 & 0 & 0 & 0 & 0 & 0 & 0 & 0 & 0 & 0 & 1 \end{array}\right)$$

#### 4.1.3. Partitioning the Reachability Matrix and the Development of a Digraph

Through the hierarchical division of the reachability matrix, cognitive attributes can be categorized. The first step is to create a reachability set *R* (S_i_) from all elements with a value of 1 in the i-th row of the reachability matrix *R*; the second step is to define all attributes with a value of 1 in the i-th column of the reachability matrix *R* as the antecedent set *A* (S_i_); the third step is to define the intersection *R* (S_i_) ∩ *A* (S_i_) of the reachability set and the antecedent set as an intersection set. When the elements in the intersection set are the same as in the reachability set, a hierarchical extraction of the elements can be carried out. The elements extracted in the first batch are the highest-level element set L1 (see Table 3). Subsequently, the elements in L1 are deleted to obtain a new reachability matrix R2, and the previous procedure is repeated until all levels of the structure are modeled. In the present case, the 16 variables, along with their reachability set, antecedent set, and intersection set, are presented in Table 3.

**Figure 2:**
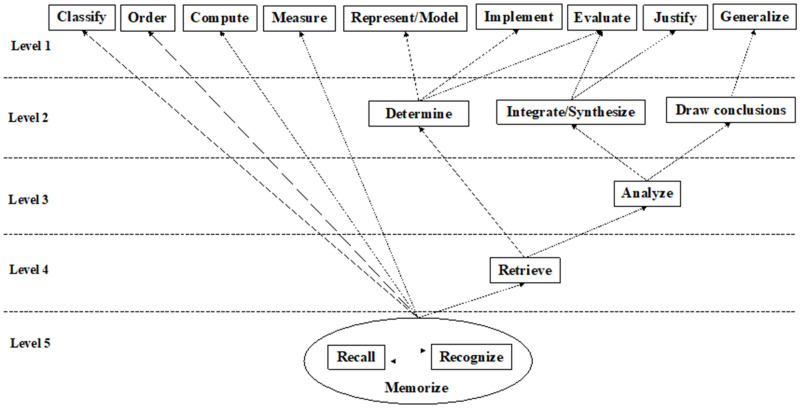
Initial mathematics cognitive model.

According to this hierarchical extraction method, a cognitive attribute model with five levels was finally formed, of which the first level included Classify S3, Order S4, Compute S5, Measure S7, Represent/Model S9, Implement S10, Evaluate S13, Generalize S15, and Justify S16. The second level included Determine S8, Integrate/synthesize S12 and Draw Conclusions S14. The third level had Analyze S11; the fourth level had Retrieve S6; Level 5 included Recall S1 and Recognize S2. According to the stratification results, a mathematical cognitive model can be drawn, as shown in Figure 2. Among them, Recall and Recognize formed a ring, indicating that there is a high degree of connection between them. Combined with the students’ actual learning situation and the expert opinions, these two attributes can be combined into one attribute—“Memorize”.

### 4.2. Revision and Verification of the Cognitive Model: Integrating the Three Domains of Cognition

**Figure 3:**
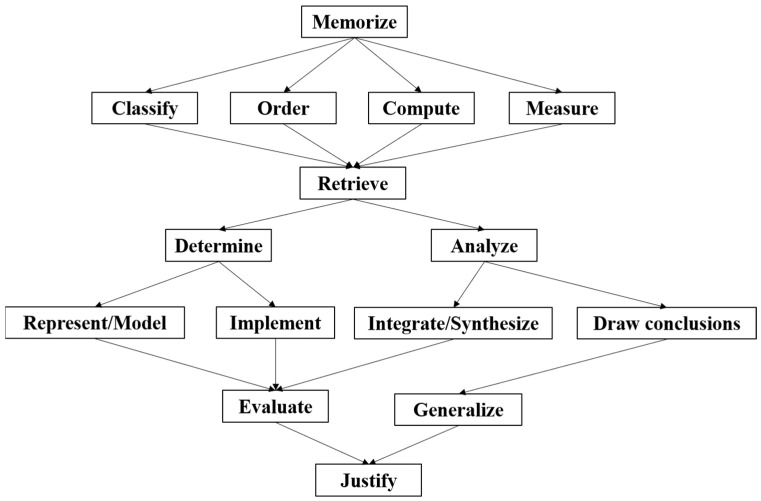
Final mathematics cognitive model.

Through the ISM method, a five-level cognitive model was preliminarily formed in this study. Based on the cognitive framework theory of the TIMSS and the expert group interviews, the preliminary model was modified, and the revised model is illustrated in Figure 3, below.

The reasons for the adjustment are as follows: first, the cognitive framework of the TIMSS consists of three domains: “knowing”, “application” and “reasoning”, and there is a progressive relationship between them. Therefore, the preliminary cognitive model can be integrated regarding the three domains. Second, experts proposed that “in complex problems, attributes such as classification, sorting, measurement, operation” are also related to other attributes and should be considered to integrate them with other attributes. Thirdly, based on the cognitive field in Bloom’s taxonomy, “knowing, applying and reasoning” has a progressive relationship from low to high cognitive requirements.

**Figure 4:**
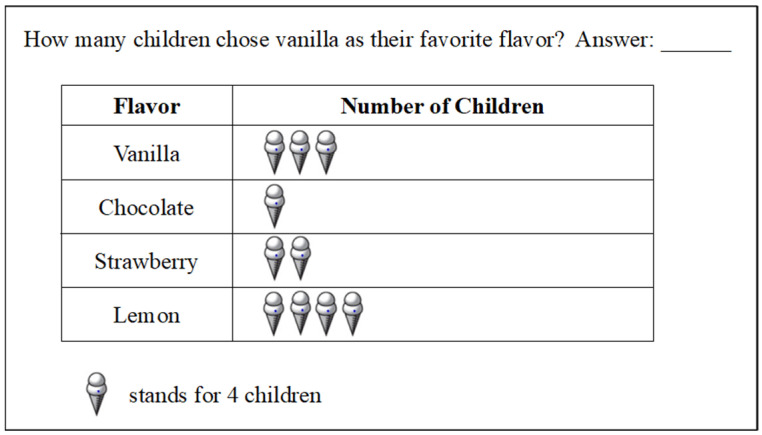
TIMSS sample problem 1.

To assess the model’s validity, a fourth-grade student was randomly selected to provide verbal reports on the TIMSS items. Taking the Item in Figure 4 as an example, the student’s oral reports were as follows: “Because one ice cream represents four children, and there are three on the vanilla ice cream, so 4 × 3 = 12 children”. The students’ solutions included paths to “retrieve information (one ice cream represents four children and three on the vanilla ice cream)”, “select strategy (multiplication is chosen)”, and “representation modeling (4 × 3 = 12 children)”.

**Figure 5:**
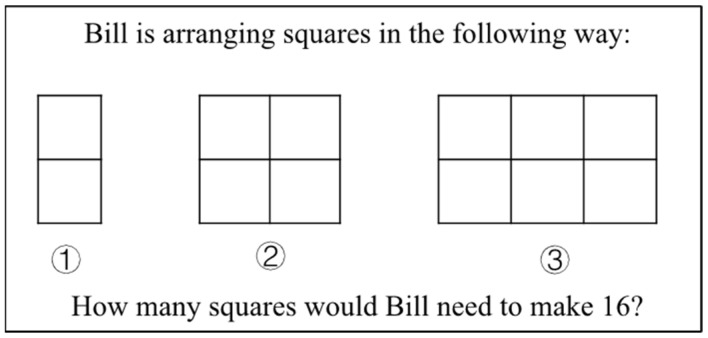
TIMSS sample problem 2.

Taking the items in Figure 5 as an example, the student’s oral report was as follows: “Picture ① has 1 column, picture ② has 2 columns, picture ③ has 3 columns, then picture ④ has 4 columns, and picture ⑤ has 5 columns, picture ⑯ has 16 columns, each column has two squares, so 16 × 2 is equal to 32 small squares”. The student first observed the information in the question and made effective inferences by analyzing the relationship between the number of columns in the picture and the number of the picture. Finally, the student summarized the 16th graph determined the number of columns (picture ⑯ has 16 columns) and obtained the answer to the question.

The revised cognitive model integrates the attributes of “knowing, applying and reasoning” from a low level to high level so that the model not only conforms to the relationship between the attributes but also has operability, which provides a theoretical basis for cognitive diagnosis. “Memorize” is the model’s starting point, and “justify” is the model’s ending point. Students can experience different paths in the process of problem solving.

## 5. Discussion

Based on the cognitive framework in the TIMSS, this study employed interpretive structural modeling to analyze the results of a survey of experts, consolidate the 16 dimensions in the TIMSS cognitive assessment into 15 attributes, and finally form a cognitive model of students’ mathematical problem solving. This cognitive model describes a variety of cognitive processes that students experience when solving mathematical problems and provides a theoretical foundation for cognitive-diagnosis-based mathematical evaluation.

### 5.1. The Validation of the Construction of the Cognitive Model

A realistic cognitive model is not only the theoretical foundation for a cognitive diagnostic assessment but it is also the premise of the cognitive diagnostic assessment. There is currently no mature approach to constructing a cognitive model, and expert methods or verbal reports are usually adopted by researchers [46]. Although these methods are relatively convenient to use, they often have disadvantages such as inconsistent conclusions, a high level of subjectivity, and difficulty in synthesizing the viewpoints of different experts. Interpretive structural modeling is a useful tool for dealing with a complex relational system: it can combine related and unrelated elements in the system into a comprehensive model [47,48,49]. In this study, the interpretative structure model was applied to construct a cognitive model which can integrate the opinions of various experts and avoid the excessive subjectivity of the model. Multiple methods were adopted in this study. Firstly, the interpretive structural model was used to integrate the data calibrated by experts, and the contribution of each expert was completely considered to clarify the relationship between attributes. Secondly, expert interviews and verbal reports were used to revise and test the validity of the cognitive model. Through an expert interview method and Bloom’s taxonomy, the model obtained from the interpretive structural model was modified to make it more consistent with the logic of mathematics education. From the perspective of students, a verbal report can visualize the students’ thinking processes and verify the cognitive model constructed. The rationality and representation of the model were ensured by the cognitive model developed using various ways. This method can be used to produce a more realistic and reasonable theoretical model not only in the production of cognitive models but also in the development of content models.

### 5.2. The Application of the Cognitive Model in Education

The cognitive model constructed in this study is composed of 15 cognitive attributes which can provide a high-precision cognitive model framework for cognitive diagnostic assessment. Cognitive models can provide guidance for educational measurement and instruction and guide test preparation and curriculum development [50]. The development of items based on a cognitive diagnosis necessitates the use of an effective cognitive theoretical framework. Mathematical problem solving is a complicated cognitive process, and only by clearly analyzing the students’ cognitive processes of problem solving can we achieve a deep understanding of students’ learning [51,52]. Teachers and students themselves can identify their mastery of each attribute through the cognitive model to make a plan for follow-up instruction and learning [53] (pp. 61–84.)

The mathematical cognitive model constructed in this paper can provide theoretical support for evaluating students’ mathematical abilities. The exam results can be used to highlight a student’s grasp of each attribute in a more defined manner, allowing future teaching activities to be better guided. Taking “retrieve “as an example, it can be seen from the model that it is a key attribute and is the basis for students to further select strategies and analysis. Students will be unable to answer mathematical questions if they are unable to extract information from multiple representations such as text and charts. PISA, PIRLS, NAEP, and other assessments all take into account the importance of international digital reading literacy [54]. Similarly, it is critical in mathematics education to assist students in comprehending the transformation between many representation systems so that mathematics may be used to address issues more effectively [55]. The current mathematics curriculum system takes the knowledge content as modules. With the gradual enrichment of research on mathematics cognition, the development of courses with cognition as the theme is also urgently needed. Mathematical modeling, for example, is a type of course distinct from algebra and geometry which has received increasing attention in recent years [56] (pp. 233–253).

The development of a reasonable mathematical cognitive model can serve as a theoretical foundation for courses, teaching, and assessment, and can facilitate individualized learning and adaptive learning systems design [57,58]. Using the establishment of a question bank as an illustration, an adaptive learning system necessitates the accumulation of an extensive repository of mathematical problems for diagnosing and evaluating students’ learning abilities, as well as for assisting in teaching. To fulfill these functions, the test questions in the question bank must be annotated with cognitive labels. The mathematical cognitive model proposed in this study provides a theoretical basis for label formulation, empowering experts to manually label the test questions according to the 15 cognitive attributes. Furthermore, leveraging the hierarchical and directional features of the cognitive model, the manually labeled tags can be automatically corrected. The task bank developed in accordance with the cognitive model constitutes a crucial component of the adaptive system and serves as the fundamental aspect of cognitive diagnostic assessment [59]. Through the labeling of the test questions’ cognitive attributes, a targeted evaluation of students’ mathematical abilities can be provided, thereby facilitating personalized assessment and personalized learning.

## 6. Limitations and Further Research

Although this study offers a new approach to constructing cognitive models, there is still a significant amount left to explore. Firstly, it is important to note that the cognitive model of mathematical cognition developed in this study was solely constructed using interpretive structural modeling (ISM) and does not incorporate specific mathematical content. However, cognitive attributes, such as procedures, skills, strategies, and knowledge, are crucial in the problem-solving process. This includes not only procedures and skills but also knowledge. The vast system of mathematical knowledge is at the heart of mathematics education, and constructing a cognitive model that integrates knowledge content requires considerable time and effort. Therefore, mathematics educators and cognitive model researchers should continue to focus their efforts on this task. Secondly, while the cognitive model was built using various research approaches, the underlying data still rely on expert calibration, and the model’s rationality must be evaluated using longitudinal data from students. The cognitive model serves as the foundation for cognitive diagnosis, and cognitive diagnostic assessments can help refine the model further. As a result, cognitive diagnostic assessments can verify the validity of the mathematical cognitive model in the following stages of research. Thirdly, it should be noted that this cognitive model is exclusively suited to the context of mathematical problem-solving processes and has limited applicability to other domains. The cognitive attributes investigated in this study, such as compute, modeling, and classify, possess distinctive mathematical characteristics that diverge from the core competencies of other disciplines. Nevertheless, it is worth highlighting that some of these cognitive attributes are also relevant to other subjects, such as retrieve and classify, which hold crucial value in science education.

However, while the generalizability of this model is limited, the procedure of constructing a cognitive model using the interpretive structural modeling (ISM) can be applied to the construction of cognitive models in other disciplines. For instance, linguistics has unique cognitive attributes, such as reading and listening, which can be hierarchically structured using the explanatory structural model to develop a cognitive model suitable for language learning. Moreover, it is imperative to investigate whether the mathematical cognitive model constructed in this study outperforms other cognitive models, which is an area that requires further exploration. Future research can focus on the efficacy of this cognitive model and comparative analyses with other cognitive models.

## Figures and Tables

**Table 1:** Cognitive attributes.

Dimensions	Attributes	Code	Connation
Knowing	Recall	S_1_	Recall definitions, terminology, number properties, units of measurement, geometric properties, and notation (e.g., S = ab; S = v × t).
	Recognize	S_2_	Recognize numbers, expressions, quantities, and shapes. Recognize entities that are mathematically equivalent (e.g., equivalent familiar fractions, decimals, and percentages).
	Classify	S_3_	Classify numbers, expressions, quantities, and shapes by common properties.
	Order	S_4_	The arrangement of numbers or other elements according to certain rules.
	Compute	S_5_	Carry out algorithmic procedures for +, –, ×, ÷, or a combination of these with whole numbers, fractions, decimals, and integers. Carry out straightforward algebraic procedures.
	Retrieve	S_6_	Retrieve information from graphs, tables, texts, or other sources.
	Measure	S_7_	Use measuring instruments and choose appropriate units of measurement.
Applying	Determine	S_8_	Determine efficient/appropriate operations, strategies, and tools for solving problems for which there are commonly used methods of solution.
	Represent/ Model	S_9_	Display data in tables or graphs; create equations, inequalities, geometric figures, or diagrams that model problem situations; and generate equivalent representations for a given mathematical entity or relationship.
	Implement	S_10_	Implement strategies and operations to solve problems involving familiar mathematical concepts and procedures.
Reasoning	Analyze	S_11_	Determine, describe, or use relationships among numbers, expressions, quantities, and shapes.
	Integrate/ Synthesize	S_12_	Link different elements of knowledge, related representations, and procedures to solve problems.
	Evaluate	S_13_	Evaluate alternative problem-solving strategies and solutions.
	Draw Conclusions	S_14_	Make valid inferences on the basis of information and evidence.
	Generalize	S_15_	Make statements that represent relationships in more general and more widely applicable terms.
	Justify	S_16_	Provide mathematical arguments to support a strategy or solution.

**Table 2:** Structural self-interactive matrix (SSIM).

O	O	O	O	O	O	O	O	O	V	V	V	V	V	X	S1
O	O	O	O	O	O	O	O	O	V	V	O	V	V	S2	
O	O	O	O	O	O	O	O	O	O	O	O	O	S3		
O	O	O	O	O	O	O	O	O	O	O	O	S4			
O	O	O	O	O	O	O	O	O	O	O	S5				
O	O	V	O	V	V	V	V	V	O	S6					
O	O	O	O	O	O	O	O	O	S7						
O	O	O	V	O	O	V	V	S8							
O	O	O	O	O	O	O	S9								
O	O	O	O	O	O	S10									
V	V	V	V	V	S11										
V	O	O	V	S12											
O	O	O	S13												
O	V	S14													
O	S15														
S16															

**Table 3:** The first level of mathematical cognitive attributes.

S_i_	*R* (S_i_)	*A* (S_i_)	*R* (S_i_) ∩ *A* (S_i_)	Level
1	1,2,3,4,5,6,7,8,9,10,11,12,13,14,15,16	1,2	1,2	
2	1,2,3,4,5,6,7,8,9,10,11,12,13,14,15,16	1,2	1,2	
3	3	1,2,3	3	1
4	4	1,2,4	4	1
5	5	1,2,5	5	1
6	6,8,9,10,11,12,13,14,15,16	1,2,6	6	
7	7	1,2,7	7	1
8	8,9,10,13	1,2,6,8	8	
9	9	1,2,6,8,9	9	1
10	10	1,2,6,8,10	10	1
11	11,12,13,14,15,16	1,2,6,11	11	
12	12,13,16	1,2,6,11,12	12	
13	13	1,2,6,8,11,12,13	13	1
14	14,15	1,2,6,11,14	14	
15	15	1,2,6,11,14,15	15	1
16	16	1,2,6,11,12,16	16	1

## Data Availability

The datasets generated and/or analyzed during the current study are available from the corresponding author upon reasonable request.

## References

[1] Schoevers E.M., Leseman P.P., Slot E.M., Bakker A., Keijzer R., Kroesbergen E.H. (2019). Promoting pupils’ creative thinking in primary school mathematics: A case study. Think. Ski. Creat..

[2] Firdaus F., Kailani I., Bakar M.N.B., Bakry B. (2015). Developing critical thinking skills of students in mathematics learning. J. Educ. Learn. EduLearn.

[3] Peter E.E. (2012). Critical thinking: Essence for teaching mathematics and mathematics problem solving skills. Afr. J. Math. Comput. Sci. Res..

[4] Cook K.L., Bush S.B. (2018). Design thinking in integrated STEAM learning: Surveying the landscape and exploring exemplars in elementary grades. Sch. Sci. Math..

[5] Bolt D. (2007). The present and future of IRT-based cognitive diagnostic models (ICDMs) and related methods. J. Educ. Meas..

[6] DiBello L.V., Stout W. (2007). Guest editors’ introduction and overview: IRT-based cognitive diagnostic models and related methods. J. Educ. Meas..

[7] Mislevy R.J. (1989). Foundations of a New Test Theory.

[8] Leighton J.P., Gierl M.J. (2007). Defining and evaluating models of cognition used in educational measurement to make inferences about examinees’ thinking processes. Educ. Meas. Issues Pract..

[9] Zhang S., Chang H.H. (2016). From smart testing to smart learning: How testing technology can assist the new generation of education. Int. J. Smart Technol. Learn..

[10] Walkington C., Bernacki M.L. (2020). Appraising research on personalized learning: Definitions, theoretical alignment, advancements, and future directions. J. Res. Technol. Educ..

[11] Rupp A.A., Templin J.L. (2008). Unique Characteristics of Diagnostic Classification Models: A Comprehensive Review of the Current State-of-the-Art. Meas. Interdiscip. Res. Perspect..

[12] Henson R.A., Templin J.L., Willse J.T. (2009). Defining a family of cognitive diagnosis models using log-linear models with latent variables. Psychometrika.

[13] Templin J.L., Henson R.A. (2006). Measurement of psychological disorders using cognitive diagnosis models. Psychol. Methods.

[14] Nichols P.D. (1994). A framework for developing cognitively diagnostic assessments. Rev. Educ. Res..

[15] DiBello L.W., Stout W.F., Roussos L.A., Nichols P.D., Chipman S.F., Brennan R.L. (1995). Unified cognitive/psychometric diagnosis assessment likelihood-based classification techniques. Cognitively Diagnostic Assessment.

[16] Gierl M.J., Alves C. (2010). Using the attribute hierarchy method to make diagnostic inferences about examinees’ knowledge and skills in mathematics: An operational implementation of cognitive diagnostic assessment. Int. J. Test..

[17] Siegler R.S., Gershkoff-Stowe L., Rakison D. (2005). Models of categorization: What are the limits?. Building Object Categories in Developmental Time.

[18] Leighton J.P., Gierl M.J. (2007). Cognitive Diagnostic Assessment for Education: Theory and Applications.

[19] Pugh D., De Champlain A., Gierl M., Lai H., Touchie C. (2016). Using cognitive models to develop quality multiple-choice questions. Med. Teach..

[20] Gierl M.J. (2007). Making diagnostic inferences about cognitive attributes using the rule-space model and attribute hierarchy method. J. Educ. Meas..

[21] Leighton J.P., Cui Y., Cor M.K. (2009). Testing expert-based and student-based cognitive models: An application of the attribute hierarchy method and hierarchy consistency index. Appl. Meas. Educ..

[22] Dogan E., Tatsuoka K. (2008). An international comparison using a diagnostic testing model: Turkish students’ profile of mathematical skills on TIMSS-R. Educ. Stud. Math..

[23] Battista M.T. (2004). Applying cognition-based assessment to elementary school students’ development of understanding of area and volume measurement. Math. Think. Learn..

[24] Leighton J.P., Gierl M.J., Hunka S.M. (2004). The attribute hierarchy method for cognitive assessment: A variation on Tatsuoka’s rule-space approach. J. Educ. Meas..

[25] Wu X., Wu R., Chang H.H., Kong Q., Zhang Y. (2020). International Comparative Study on PISA Mathematics Achievement Test Based on Cognitive Diagnostic Models. Front. Psychol..

[26] Mullis I.V.S., Martin M.O., Foy P. (2005). IEA’s TIMSS 2003 International Report on Achievement in the Mathematics Cognitive Domains: Findings from a Developmental Project.

[27] Mullis I.V.S., Martin M.O., Ruddock G.J., Sullivan C.Y., Preuschoff C. (2009). TIMSS 2011 Assessment Frameworks.

[28] Macnab D. (2000). Raising standards in mathematics education: Values, vision, and TIMSS. Educ. Stud. Math..

[29] Mullis I.V.S., Martin M.O. (2014). TIMSS Advanced 2015 Assessment Frameworks. http://timssandpirls.bc.edu/timss2015-advanced/frameworks.html.

[30] Krathwohl D.R. (2002). A revision of Bloom’s taxonomy: An overview. Theory Pract..

[31] Thompson T. (2008). Mathematics teachers’ interpretation of higher-order thinking in Bloom’s taxonomy. Int. Electron. J. Math. Educ..

[32] Crowe A., Dirks C., Wenderoth M.P. (2008). Biology in bloom: Implementing Bloom’s taxonomy to enhance student learning in biology. CBE-Life Sci. Educ..

[33] Lee Y.S., Park Y.S., Taylan D. (2011). A cognitive diagnostic modeling of attribute mastery in Massachusetts, Minnesota, and the U.S. national sample using the TIMSS 2007. Int. J. Test..

[34] Attri R., Dev N., Sharma V. (2013). Interpretive structural modelling (ISM) approach: An overview. Res. J. Manag. Sci..

[35] Raj T., Attri R. (2011). Identification and modeling of barriers in the implementation of TQM. Int. J. Prod. Qual. Manag..

[36] Raj T., Shankar R., Suhaib M. (2008). An ISM approach for modelling the enablers of flexible manufacturing system: The case for India. Int. J. Prod. Res..

[37] Sarikhani Y., Shojaei P., Rafiee M., Delavari S. (2020). Analyzing the interaction of main components of hidden curriculum in medical education using interpretive structural modeling method. BMC Med. Educ..

[38] Debnath R.M., Shankar R. (2012). Improving service quality in technical education: Use of interpretive structural modeling. Qual. Assur. Educ..

[39] Ding S., Mao M., Wang W., Luo F., Cui Y. (2012). Evaluation of consistency between educational cognitive diagnostic tests and cognitive models. Acta Psychol. Sin..

[40] Mahajan R., Agrawal R., Sharma V., Nangia V. (2016). Analysis of challenges for management education in india using total interpretive structural modelling. Qual. Assur. Educ..

[41] Awuzie B.O., Abuzeinab A. (2019). Modelling Organisational Factors Influencing Sustainable Development Implementation Performance in Higher Education Institutions: An Interpretative Structural Modelling (ISM) Approach. Sustainability.

[42] Junker B.W., Sijtsma K. (2001). Cognitive assessment models with few assumptions, and connections with nonparametric item response theory. Appl. Psychol. Meas..

[43] Im S., Park H.J. (2010). A comparison of US and Korean students’ mathematics skills using a cognitive diagnostic testing method: Linkage to instruction. Educ. Res. Eval..

[44] Choi K.M., Lee Y.S., Park Y.S. (2015). What CDM can tell about what students have learned: An analysis of TIMSS eighth grade mathematics. Eurasia J. Math. Sci. Technol. Educ..

[45] Akbay L., Terzi R., Kaplan M., Karaaslan K.G. (2017). Expert-Based Attribute Identification and Validation on Fraction Subtraction: A Cognitively Diagnostic Assessment Application. J. Math. Educ..

[46] Loibl K., Leuders T., Dörfler T. (2020). A framework for explaining teachers’ diagnostic judgements by cognitive modeling (DiaCoM). Teach. Teach. Educ..

[47] Yadav D.K., Barve A. (2015). Analysis of critical success factors of humanitarian supply chain: An application of Interpretive Structural Modeling. Int. J. Disaster Risk Reduct..

[48] Poduval P.S., Pramod V.R., VP J.R. (2015). Interpretive structural modeling (ISM) and its application in analyzing factors inhibiting implementation of total productive maintenance (TPM). Int. J. Qual. Reliab. Manag..

[49] Lim M.K., Tseng M.L., Tan K.H., Bui T.D. (2017). Knowledge management in sustainable supply chain management: Improving performance through an interpretive structural modelling approach. J. Clean. Prod..

[50] Cheng Y. (2009). When cognitive diagnosis meets computerized adaptive testing. Psychometrika.

[51] DeCarlo L.T. (2011). On the analysis of fraction subtraction data: The DINA model, classification latent class sizes, and the Q-matrix. Appl. Psychol. Meas..

[52] Laurens T., Batlolona F.A., Batlolona J.R., Leasa M. (2017). How does realistic mathematics education (RME) improve students’ mathematics cognitive achievement?. Eurasia J. Math. Sci. Technol. Educ..

[53] Norris S.P., Macnab J.S., Phillips L.M., Leighton J.P., Gierl M.J. (2007). Cognitive Modeling of Performance on Diagnostic Achievement Tests A Philosophical Analysis and Justification. Cognitively Diagnostic Assessment for Education: Theory and Applications.

[54] Wang Z., Wan P., Nan X., Liu C. (2020). Enhancing Digital Reading Literacy: Multidimensional Comparison and Its Enlightenment of International Digital Reading Literacy Evaluation Indexes. Open Educ. Res..

[55] Richardson K., Berenson S., Staley K. (2009). Prospective elementary teachers use of representation to reason algebraically. J. Math. Behav..

[56] Brown J.P., Ikeda T. (2019). Conclusions and Future Lines of Inquiry in Mathematical Modelling Research in Education.

[57] Yang T.C., Hwang G.J., Yang S.J.H. (2013). Development of an adaptive learning system with multiple perspectives based on students’ learning styles and cognitive styles. J. Educ. Technol. Soc..

[58] Chen S., Zhang J. The adaptive learning system based on learning style and cognitive state. Proceedings of the 2008 International Symposium on Knowledge Acquisition and Modeling.

[59] Pei X., Yang S., Huang J., Xu C. Self-Attention Gated Cognitive Diagnosis for Faster Adaptive Educational Assessments. Proceedings of the 2022 IEEE International Conference on Data Mining (ICDM).

